# From Distraction to Delay: Unpacking Academic Procrastination

**DOI:** 10.5964/ejop.19203

**Published:** 2026-08-28

**Authors:** Rosa Angela Fabio, Miriam Ricciardello, Giulia Picciotto

**Affiliations:** 1 Department of Biomedical, Morphological and Functional Imaging Sciences, University of Messina, Messina, Italy; 2 Department of Cognitive Sciences, Psychological, Educational, and Cultural Studies, University of Messina, Messina, Italy; 3 Department of Clinical and Experimental Medicine, University of Messina, Messina, Italy; Stanford University, Stanford, USA

**Keywords:** procrastination, social media addiction, personality, need for achievement, inattention, hyperactivity, psychological well-being

## Abstract

Academic procrastination undermines students’ academic performance and psychological well-being. This study investigated key psychological and cognitive factors associated with procrastination, including personality traits, need for achievement, inattention, hyperactivity, and social media addiction. A total of 464 Italian university students completed validated self-report questionnaires; a subsample of 80 participants also completed cognitive tasks assessing attention, inhibitory control, and working memory. Predictors of procrastination were examined using multiple regression and a path analysis model (structural equation modeling). Exploratory statistical indirect-effect analyses were also conducted; given the cross-sectional design, these were interpreted as associational rather than causal. Procrastination was negatively associated with conscientiousness and need for achievement, and positively associated with inattention, hyperactivity, and problematic social media use, with inattention emerging as the strongest statistical correlate. Importantly, the integration of self-report and objective performance data revealed that high procrastinators showed poorer executive functioning. These findings highlight the combined role of personal and cognitive factors in academic procrastination. Interventions aimed at enhancing attentional control and strengthening need for achievement may help reduce procrastination and improve students’ academic outcomes and well-being.

Academic procrastination, defined as the voluntary delay of important tasks despite expecting negative consequences, is a widespread self-regulatory failure among students, often accompanied by feelings of guilt, stress, and reduced well-being (Klingsieck et al., 2013; Sirois & Pychyl, 2016). Some scholars have proposed that procrastination may at times function as a short-term emotion-regulation strategy (Huang et al., 2025; Sirois & Pychyl, 2016). However, the majority of research supports its maladaptive impact on academic performance, cognitive functioning, and psychological health (Steel, 2007; Scheunemann et al., 2022). Numerous studies have examined the individual predictors of procrastination, yet these are often investigated in isolation, without integrating them into comprehensive, theory-driven models.

The Temporal Motivation Theory (TMT; Steel & König, 2006) offers a valuable framework to explain procrastination as the outcome of both personality dispositions and contextual influences. According to TMT, the perceived utility of a task increases with expectancy and value, and decreases with impulsiveness and delay, thereby influencing motivation and task engagement (Steel & König, 2006). It emphasizes the importance of attentional control and goal-maintenance mechanisms, which, when impaired, can lead to reduced task initiation and greater susceptibility to distractions (Steel et al., 2018; Netzer Turgeman & Pollak, 2023).

Consistent with TMT, previous research has demonstrated that inattention and hyperactivity (core features of executive dysfunction) are robust factors associated with procrastination. These symptoms are associated with difficulties in sustained attention, resistance to distraction, and goal-directed behavior (Wiwatowska et al., 2025; Sönmez et al., 2023; Oguchi et al., 2021).

Although these studies do not examine procrastination directly, behavioral and neurocognitive research on attentional control indicates that reduced allocation of attentional resources and impairments in executive functioning may contribute to difficulties in goal-directed behavior relevant to procrastination (Aristodemou et al., 2024; Ghani et al., 2020; Michałowski et al., 2020). Neuroimaging studies further support this view, showing reduced volume and activation in prefrontal areas involved in cognitive control (Chen et al., 2020; Hu et al., 2018). In parallel, personality traits, particularly low conscientiousness, have consistently been linked to procrastination (Steel, 2007; Koppenborg & Klingsieck, 2022). Conscientiousness reflects self-discipline, goal-orientation, and impulse control, all of which protect against procrastination. However, less is known about the role of need for achievement, defined as an individual’s drive to attain competence and success. Need for achievement reflects both a desire to achieve (hope for success) and to avoid failure (fear of failure), and it has been associated with increased task engagement and lower procrastination in academic contexts (Elliot & Church, 1997; Senécal et al., 1995). Despite its theoretical relevance, this variable has received less empirical attention in recent integrative models of procrastination (Steel, 2010; Sirois, Yang, & van Eerde, 2019; Koppenborg & Klingsieck, 2022).

A further dimension gaining attention is problematic social media use, particularly among university students. Social media platforms offer immediate gratification and serve as potent distractors from goal-directed activities (Abd Ellatif Elsayed, 2025; Barton et al., 2021; Fabio et al., 2022; Przepiorka et al., 2023).

Excessive use has been linked to attentional lapses, poorer academic performance, and increased procrastination (Hammad & Awed, 2023; Fabio & Urso, 2014; Fabio & Tripodi, 2024). These findings suggest that social media may hijack attentional control systems, reinforcing avoidance behaviors and displacing cognitively demanding tasks. While each of these factors (personality, need for achievement, executive attention, and media use) has been studied in relation to procrastination, few studies have examined them in combination. This fragmented approach limits our understanding of how multiple individual and environmental factors jointly contribute to procrastination.

Moreover, psychological well-being, although frequently discussed as an outcome of procrastination, is rarely integrated into formal models of its predictors, despite growing evidence of a bidirectional relationship (Sirois & Pychyl, 2016). To address these gaps, the present study tests a theory-driven path analysis model integrating key predictors of academic procrastination: personality traits (including conscientiousness and neuroticism), need for achievement, symptoms of inattention and hyperactivity, problematic social media use, and executive functioning (attentional capacity, inhibition, and working memory). Psychological well-being was also included as a distal outcome, to examine whether procrastination statistically accounted for associations between self-regulatory capacities and subjective well-being. By combining self-report data with objective cognitive measures in a large sample of university students, this study aims to provide a more comprehensive understanding of the interrelated mechanisms underlying procrastination.

We hypothesized that: (1) Need for achievement and conscientiousness would be negatively associated with procrastination; (2) Inattention, hyperactivity, and problematic social media use would positively predict procrastination; (3) Procrastination would be negatively associated with psychological well-being; (4) Individuals with higher levels of procrastination would show poorer performance on executive tasks, particularly those involving attention and inhibition.

This integrated model seeks to clarify the multidimensional nature of academic procrastination and inform more targeted interventions aimed at improving students’ academic engagement and mental health.

## Method

### Participants

The study involved a total of 464 university students (185 men, 40.09%; 277 women, 59.91%; 2 non-binary, 0.2%) recruited from multiple Italian universities. Participants ranged in age from 18 to 60 years (*M* = 24.97, *SD* = 7.36). Recruitment was carried out through various online channels, including social media platforms, university mailing lists, and community forums, using announcements that described the study’s aims and procedures. The distribution of students across academic disciplines is presented in Table 1.

**Table 1:** Demographic Statistics of Participants (n = 464)

Measures	Frequency (*n*)	Percentage (%)	*M* (*SD*)
Gender			
Female	277	59.91	
Male	185	40.09	
Non-binary	2	0.2	
Age			24.97 (7.36)
Area of study			
Scientific and technological	64	14.10	
Legal and economic	42	9.25	
Social and communication	53	11.68	
Psychological and educational	239	52.64	
Humanistic and literary	20	4.41	
Artistic	36	7.93	

*Note. M* = Means; *SD* = Standard Deviation.

A priori power analysis was not conducted; however, given reviewer feedback, we conducted a sensitivity analysis using *G*Power 3.1* (Faul et al., 2009) to estimate the smallest effect size that could be reliably detected with the available sample size. For bivariate correlations, with α = .05 and power (1−β) = .80, the full sample of *N* = 464 allows detection of small-to-moderate effect sizes (*r* ≥ .13). For regression or path models, this sample provides sufficient power to detect small-to-moderate effects (*f^2^* ≥ .03) with up to 10 predictors. Thus, the study is adequately powered to detect effects of practical relevance, in line with those commonly observed in psychological research. A subsample of 80 participants completed the computerized cognitive tasks. A sensitivity analysis for this subset indicates that, with α = .05 and power = .80, the minimum detectable effect is *r* = .31 for correlational analyses (two-tailed), corresponding to a medium effect size (Cohen, 1988). Therefore, findings based on this smaller sample should be interpreted with caution, particularly where effect sizes are below this threshold.

### Materials

In the present study, questionnaires were administered to assess personality traits, need for achievement, inattention, procrastination, and social media addiction.

#### The Big Five Inventory–10 (BFI-10)

Personality traits were assessed using the Italian version of the 10-item Big Five Inventory (BFI-10; Guido et al., 2015). It is a 10-item questionnaire developed to assess the five major dimensions of personality (Rammstedt & John, 2007). Each item is rated on a 5-point Likert scale, ranging from “strongly disagree” to “strongly agree.” The BFI-10 is a shortened version of the original Big Five Inventory (BFI-44), developed by John, Donahue, & Kentle (1991), and was designed to provide a brief yet effective measure of personality traits in large-scale surveys or time-constrained settings. The Cronbach’s alpha coefficient is 0.81.

#### Need for Achievement Questionnaire (NAQ)

The need for achievement was assessed using the corresponding subscale of the NAQ (Heckert et al., 2000). This subscale includes 5 items specifically targeting individuals’ desire to excel and improve previous performance. Participants rated their agreement on a 5-point Likert scale ranging from 1 (strongly disagree) to 5 (strongly agree). In the present study, the scale demonstrated good internal consistency, with Cronbach’s alpha coefficient of 0.77.

#### World Health Organization Five Well-Being Index (WHO-5)

The World Health Organization Five Well-Being Index (WHO-5; World Health Organization, 1998) is a self-report measure composed of five items assessing perceived psychological well-being. Items are rated on a 6-point Likert scale ranging from 0 (never) to 5 (always) and cover dimensions such as positive mood, vitality, and interest in daily activities. Higher scores indicate greater levels of well-being. In the present study, the Italian version of the WHO-5 was used, which has demonstrated good cross-cultural validity, acceptable scalability (Loevinger’s coefficient of homogeneity *H* = 0.61), and unidimensionality, with fewer than 5% of *t*-tests reaching significance (Carrozzino et al., 2022).

#### Adult ADHD Self-Report Scale (ASRS)

For the assessment of symptoms of inattention, hyperactivity, and impulsivity related to ADHD, the Adult Self-Report Scale (ASRS; Kessler et al., 2005; Somma et al., 2019) was used. This scale consists of 18 items that assess the core symptoms of ADHD, divided into two dimensions: inattention and hyperactivity/impulsivity. Responses are given on a 5-point Likert scale, ranging from 0 (never) to 4 (very often). The ASRS has demonstrated good psychometric properties, with validity and reliability supported by studies conducted on both clinical samples and the general population (Gray et al., 2014). Internal consistency, as measured by Cronbach’s alpha coefficient, showed a value of α = 0.88. Furthermore, the Italian version of the scale was translated and validated by Somma et al. (2019).

#### Pure Procrastination Scale (PPS)

The Pure Procrastination Scale (PPS; Steel, 2010) consists of 12 items rated on a 5-point Likert scale ranging from 1 (very seldom/not true of me) to 5 (very often/true of me). The PPS includes a combination of subscales measuring different aspects of procrastination, with the most relevant being decisional delay (Items 1–3), implemental delay (Items 4–8), and lateness/timeliness (Items 9–12) (Svartdal & Steel, 2017). In the present study, the PPS showed a Cronbach’s alpha of .85, indicating good reliability for the current sample.

#### Bergen Social Media Addiction Scale (BSMAS)

The Bergen Social Media Addiction Scale (BSMAS; Andreassen et al., 2016) consists of six items based on the six core components of addiction—salience, mood modification, tolerance, withdrawal, conflict, and relapse—originally proposed by Griffiths (2000) to assess social media addiction. The items explore social media use over the past year and are rated on a five-point Likert scale ranging from 1 (very rarely) to 5 (very often). Higher scores indicate greater symptom severity. In the present study, the Italian version of the scale (Monacis et al., 2017) was used, which showed good internal reliability (Cronbach’s α = .88).

### Procedure

The study was reviewed and approved by the Ethics Committee of the University of Messina and was conducted in accordance with the ethical principles outlined in the Declaration of Helsinki. Participation was entirely voluntary, and no compensation or incentives were offered. All participants received a detailed explanation of the study’s objectives, expected duration, and procedures, and provided written informed consent prior to participation. The study was conducted in two distinct phases. In the first phase, participants completed a battery of self-report questionnaires administered online via the Google Forms platform.

These questionnaires assessed personality traits, need for achievement, inattention, procrastination, and social media addiction. All participants completed the Pure Procrastination Scale (PPS; Steel, 2010) to measure their tendency to procrastinate. Based on PPS scores, a subsample of 80 students was selected and divided into two groups of 40 participants each: high procrastinators (HP) and low procrastinators (LP). Group assignment was based on scores falling within the upper and lower quartiles of the PPS distribution, in order to maximize the contrast between the two experimental conditions. In the second phase, participants from both groups took part in individual in-person experimental sessions conducted in a controlled laboratory environment. Prior to the cognitive testing, standardized instructions were provided to ensure proper understanding of the tasks. The tasks, administered via the online platform “Cognitive Fun”, included assessments of attention, inhibitory control, and working memory. Each session was conducted individually and lasted approximately 30 minutes.

### Cognitive Assessment

Based on PPS scores, a sample of 80 students was selected and divided into two groups of 40 participants each: high procrastinators (HP) and low procrastinators (LP). Both groups completed a battery of cognitive tasks aimed at assessing attentional capacity, inhibitory control, and working memory. The order of task administration was randomized to control for sequence effects. Attention was examined with the Go/No-Go test by calculating the visual reaction time needed to provide the correct response and to suppress an incorrect impulsive action to 10 visual stimuli. The Flanker test is an interference task where different inputs compete with the target, thus slowing down response speed. This is a basic variant using arrows, in which subjects must identify the direction of the central arrow. The estimated completion time was less than 1 minute per session. Arrows were displayed in congruent or incongruent forms. For example, in one case, the central arrow pointed to the right, so participants had to press the right arrow key on the keyboard. In another case, the central arrow pointed to the left, so subjects had to press the left arrow key. The task consisted of 20 trials, which randomly included both congruent and incongruent forms.

Finally, the N-back task was used to assess working memory, originally introduced by Kirchner (1958) as a visuospatial task with four levels (“0-back” to “3-back”) and by Mackworth (1959) as a visual letter task with up to six levels of load. In the present study, 2-back tasks were continuous recognition measures presenting sequences of stimuli (20 images) for each item in the sequence. Subjects judged whether the current stimulus matched the one presented two trials earlier by pressing a computer key. Each stimulus was presented for a maximum of 2000 milliseconds, and a new stimulus appeared every 2500 milliseconds.

### Statistical Analysis

All analyses were conducted using SPSS (Version 28; IBM Corp.) and AMOS (Version 28; IBM Corp.) for path analysis. Descriptive statistics, including means, standard deviations, skewness, and kurtosis, as well as Pearson correlations, were first computed to assess bivariate relationships among the study variables. To examine the statistical associations between psychological traits and procrastination, we performed multiple linear regressions, including conscientiousness, inattention, hyperactivity, need for achievement, and social media addiction as predictors. A bootstrap-based indirect-effect analysis (5,000 resamples) was conducted to test whether need for achievement statistically accounted for the association between conscientiousness and procrastination. Because the data are cross-sectional, these indirect effects were interpreted as statistical rather than causal. In addition, path analysis (structural equation modeling) was employed to examine the direct and indirect associations among the predictors, procrastination, and psychological well-being. Model fit was evaluated using the chi-square statistic (χ^2^), the Comparative Fit Index (CFI, with values greater than .95 indicating good fit), the Root Mean Square Error of Approximation (RMSEA, with values less than .06 indicating good fit), and the Standardized Root Mean Square Residual (SRMR, with values less than .08), following the guidelines by Hu and Bentler (1999).

Furthermore, independent samples *t*-tests were used to compare high versus low procrastinators on executive functioning measures, and Bonferroni correction was applied for multiple comparisons. Before conducting regression and path analysis, all relevant statistical assumptions were tested. Regarding normality, skewness and kurtosis values for all continuous variables fell within the acceptable range of ±2, suggesting an approximately normal distribution (George & Mallery, 2010). Additionally, Q–Q plots and Shapiro–Wilk tests conducted on a random 10% subsample confirmed the normality of residuals.

Linearity and homoscedasticity were assessed through scatterplots of standardized residuals versus predicted values, which showed no evidence of non-linearity or heteroscedasticity. Multicollinearity was evaluated using the Variance Inflation Factor (VIF), with all predictors showing VIF values below 2.5 and tolerance values above 0.4, indicating no issues with multicollinearity (Tabachnick & Fidell, 2007).

Finally, outliers and influential cases were assessed by examining standardized residuals greater than ±3. Cook’s distance and Mahalanobis distance were also computed, and no influential cases exceeded the critical thresholds, with Cook’s D values below 1.0 and *p*-values greater than .001 for Mahalanobis distance.

## Results

### Descriptive Statistics and Correlations

Descriptive statistics for the study variables are presented in Table 2. Overall, the sample showed moderate to high levels of agreeableness (*M* = 7.01, *SD* = 1.71) and openness to experience (*M* = 7.47, *SD* = 1.84), and elevated levels of procrastination (*M* = 33.04, *SD* = 12.39), inattention (*M* = 25.19, *SD* = 6.72), and hyperactivity (*M* = 21.43, *SD* = 5.14). Pearson correlations revealed significant associations among variables (Table 3). Procrastination was negatively correlated with conscientiousness (*r* = –.51, *p* < .001) and need for achievement (*r* = –.47, *p* < .001), and positively correlated with inattention (*r* = .75, *p* < .001), hyperactivity (*r* = .56, *p* < .001), and social media addiction (*r* = .47, *p* < .001). Psychological well-being showed a negative correlation with procrastination (*r* = –.45, *p* < .001).

**Table 2:** Means (M), Standard Deviations (SD), and Theoretical Ranges Of Psychological Variables

	*M* (*SD*)	Theoretical range
*Big Five*		
Extraversion	6.19 (1.71)	2–10
Agreeableness	7.01 (1.71)	2–10
Conscientiousness	6.59 (1.66)	2–10
Neuroticism	6.74 (1.84)	2–10
Openness to experience	7.47 (1.84)	2–10
*Need for achievement*	20.24 (3.57)	5–25
*Inattention*	25.19 (6.72)	9–45
*Hyperactivity*	21.43 (5.14)	8–35
*Procrastination *	33.04 (12.39)	12–60
*Psychological Well-Being *	11.47 (3.53)	0–25
*Social Media Addiction*	17.01 (5.69)	6–30

*Note.* Theoretical range indicates the scale endpoints (minimum–maximum possible values), not the observed range in the current sample.

**Table 3:** Pearson Correlations Among the Psychological Variables

Variable	1	2	3	4	5	6	7	8	9	10	11
1. PPS	—	-0.33	-.238**	-.510**	-.310**	-.098**	-.467*	.754**	.558**	-.450**	.468**
2. Extraversion		—	.067	-.003	.015	.657**	-.008	.031	.000	.080	-.029
3. Agreeableness			—	.187**	-.321**	.108*	.203**	-.227**	-.246**	.271**	-.169**
4. Conscientiousness				—	-.250**	.059	.513**	-.538**	-.412**	.255**	-.281**
5. Neuroticism					—	-.013	-.171**	.372**	.411**	-.416**	.218**
6. Openness to experience						—	.152**	-.036	-.010	.097*	-.136**
7. Need for achievement							—	-.408**	-.278**	.219**	-.239**
8. Inattention								—	.733**	-.425**	-.471**
9. Hyperactivity									—	-.425**	.398**
10. Psychological Well-Being										—	-.345**
11. Social Media Addiction											—

*p < .05. **p < .01.

### Multiple Regression and Indirect-Effect Analysis

A multiple regression analysis confirmed that inattention (β = .47, *p* < .001) and hyperactivity (β = .26, *p* < .001) were significantly positively associated with procrastination, whereas conscientiousness (β = –.38, *p* < .001) and need for achievement (β = –.29, *p* < .001) were significantly negatively associated with procrastination. A bootstrap-based indirect-effect analysis indicated that need for achievement statistically accounted for part of the association between conscientiousness and procrastination.

The indirect effect was significant (indirect effect = –0.19, 95% CI [–0.28, –0.12], *p* < .001), suggesting that lower need for achievement is associated with higher procrastination among individuals lower in conscientiousness. Given the cross-sectional design, this indirect effect should not be interpreted as evidence of causality.

### Path Analysis

**Figure 1:**
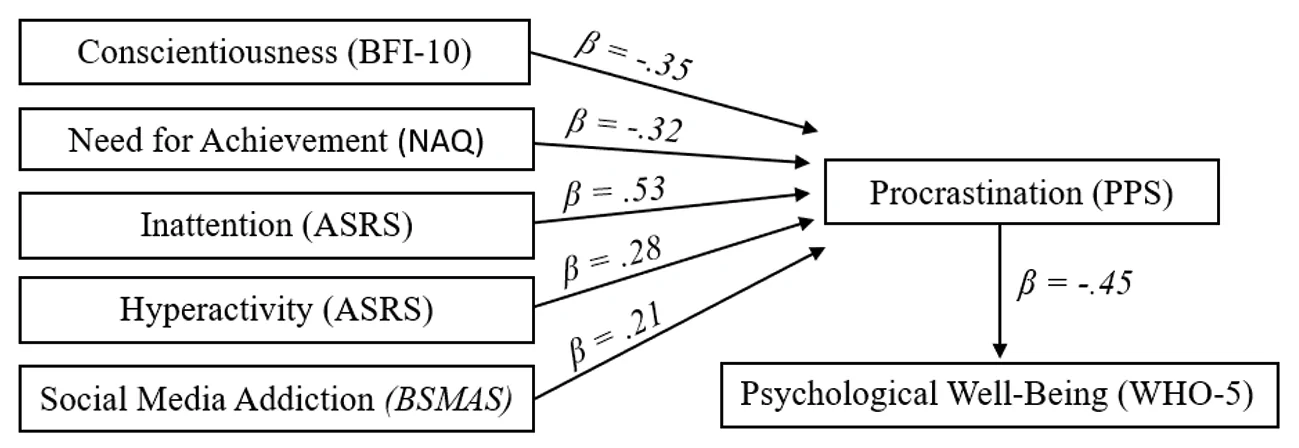
Path Analysis Model *Note.* Path analysis model illustrating the statistically significant direct relationships between psychological variables, procrastination, and psychological well-being. In line with theoretical expectations, only significant predictors are included. Conscientiousness and need for achievement are negatively associated with procrastination, while inattention, hyperactivity, and social media addiction are positively associated. Procrastination, in turn, is negatively associated with psychological well-being. Standardized beta coefficients (β) are shown along each path; all paths are significant at *p* < .001.

A structural equation model was tested to examine the direct and indirect relationships among personality traits, attentional difficulties, need for achievement, procrastination, and psychological well-being. The model demonstrated a good fit to the data: χ^2^(7) = 12.63, *p* = .08; CFI = .99; RMSEA = .038 [90% CI: .00, .07]; SRMR = .019. As shown in Figure 1, inattention (β = .53, *p* < .001), hyperactivity (β = .28, *p* < .001), and social media addiction (β = .21, *p* < .001) showed significant positive paths to procrastination. Conversely, conscientiousness (β = –.35, *p* < .001) and need for achievement (β = –.32, *p* < .001) had significant negative effects. Procrastination, in turn, was significantly associated with lower psychological well-being (β = –.45, *p* < .001), consistent with the hypothesized indirect association modeled through procrastination, although no causal inference is warranted.

Openness and agreeableness were excluded from the final model due to non-significant paths.

### Group Comparison: Executive Functioning

Participants in the highest and lowest quartiles of the procrastination distribution were classified as high procrastinators (HP; *n* = 40) and low procrastinators (LP; *n* = 40), respectively. Independent samples *t*-tests revealed that HP participants demonstrated significantly longer reaction times and lower accuracy on executive functioning tasks. In the Go/No-Go task, the average reaction time for the HP group was 695.07 milliseconds (*SD* = 188.25), compared to 584.79 milliseconds (*SD* = 96.33) for the LP group, yielding *t*(78) = –3.30, *p* = .001, and an effect size of *d* = 0.69.

In the Flanker task with incongruent stimuli, HP participants had an average reaction time of 926.60 milliseconds, whereas LP participants averaged 689.83 milliseconds, with *t*(78) = –2.92, *p* = .005, and *d* = 0.65. For the N-back task (2-back accuracy), the HP group showed a mean accuracy of .42 (*SD* = .13), while the LP group achieved a higher mean accuracy of .54 (*SD* = .24), resulting in *t*(78) = 2.68, *p* = .009, and *d* = 0.60. Additionally, there was a trend toward greater response variability among HP participants, although this difference did not reach statistical significance (*p* = .073) (see Table 4). These findings support the view that high procrastination is associated with impairments in inhibitory control, attentional stability, and working memory updating—core components of executive functioning.

**Table 4:** Differences in Cognitive Task Performance Between Low (n = 40) and High Procrastinators (n = 40)

Task	Group	*M* (*SD*)	*t* (78)	*p*	*d*
Go/No-Go (*M,* RT in ms)	Low	584.79 (96.33)	–3.30	.001	0.69
	High	695.07 (188.25)			
Go/No-Go (*SD,* RT in ms)	Low	232.02 (211.16)	–1.82	.073	0.39
	High	341.21 (315.16)			
Go/No-Go (% Correct Responses)	Low	98.00 (4.05)	0.96	.338	0.21
	High	97.00 (5.16)			
Flanker Congruent (RT in ms)	Low	652.31 (124.15)	–2.87	.005	0.64
	High	793.11 (284.26)			
Flanker Incongruent (RT in ms)	Low	689.83 (132.60)	–2.92	.005	0.65
	High	926.60 (494.91)			
Flanker (% Correct Responses)	Low	99.63 (1.33)	0.91	.364	0.20
	High	99.00 (4.11)			
N-back (RT in ms)	Low	824.93 (223.26)	–2.29	.025	0.51
	High	952.31 (272.70)			
N-back (Accuracy)	Low	0.54 (0.24)	2.68	.009	0.60
	High	0.42 (0.13)			

*Note.* RT = Reaction Time; *M* = Mean; *SD* = Standard Deviation.

## Discussion

The present study aimed to integrate multiple psychological and cognitive predictors of academic procrastination within a unified model informed by motivational principles derived from Temporal Motivation Theory (TMT; Steel & König, 2006). TMT is primarily a motivational framework and does not explicitly include personality traits or need for achievement; however, need for achievement can be considered conceptually related to task value, such that individuals with higher need for achievement may perceive academic tasks as more valuable and therefore be less likely to procrastinate. The study also provides novel contributions through the experimental assessment of executive functions, including attention and inhibitory control (Go/No-Go, Flanker tasks) and working memory (N-back task). Consistent with our hypotheses, procrastination was negatively associated with conscientiousness and need for achievement, and positively predicted by inattention, hyperactivity, and problematic social media use. Importantly, procrastination emerged as a significant intervening variable in the statistical model linking self-regulatory variables with psychological well-being, and individuals with higher procrastination scores showed significantly poorer performance in executive tasks tapping attention, inhibition, and working memory.

These findings contribute to the growing body of literature positioning procrastination as a multidimensional self-regulatory failure (Klingsieck et al., 2013; Sirois & Pychyl, 2016). While prior studies have documented the negative role of inattention (Sönmez et al., 2023; Wiwatowska et al., 2022), impulsivity (Steel et al., 2018), and low conscientiousness (Steel, 2007; Koppenborg & Klingsieck, 2022) in procrastination, this study adds to the literature by integrating these dimensions into a single path model, demonstrating their unique and joint contributions. Notably, need for achievement, despite its theoretical centrality in TMT, has received limited empirical attention in recent integrative models. Our findings highlight its protective function, statistically accounting for part of the association between conscientiousness and procrastination, in line with motivational theories emphasizing task engagement and goal orientation (Elliot & Church, 1997; Senécal et al., 1995).

A key innovation of this study lies in bridging self-report measures of procrastination with objective cognitive performance. Few studies have directly linked dispositional procrastination to behavioral indices of executive dysfunction, such as slower reaction times and reduced accuracy in Go/No-Go, Flanker, and N-back tasks. Our results show that high procrastinators exhibited impairments in inhibitory control, attentional stability, and working memory updating, consistent with prior research on executive functions, which may help explain the attentional and inhibitory difficulties observed in high procrastinators (Aristodemou et al., 2024; Michałowski et al., 2020; Ghani et al., 2020). These behavioral outcomes also align with neuroimaging findings showing reduced prefrontal activity in procrastinators (Hu et al., 2018; Chen et al., 2020), further supporting the role of executive deficits in the failure to initiate and sustain goal-directed behavior.

Importantly, by including psychological well-being as a distal outcome, this study underscores the broader personal costs of procrastination beyond academic performance. As suggested by Sirois and Pychyl (2016), procrastination not only arises from self-regulatory difficulties but also perpetuates emotional distress, guilt, and dissatisfaction—creating a feedback loop of maladaptive coping. Our model is consistent with this view, revealing that attentional and motivational deficits were statistically linked to lower well-being through higher procrastination. Because the study is cross-sectional, this pattern should be interpreted as an indirect association rather than a demonstrated causal pathway.

From an applied perspective, these findings suggest the importance of multi-level interventions targeting both dispositional traits (e.g., low conscientiousness) and cognitive control mechanisms (e.g., attention training, inhibition tasks). Moreover, curbing problematic social media use may reduce external distractions that compromise goal maintenance and delay task initiation (Przepiorka et al., 2023; Fabio & Tripodi, 2024).

### Limitations and Future Directions

Despite its strengths, this study has several limitations. Importantly, because the present study is cross-sectional, the directionality of the associations cannot be established. Although the model is theory-driven, alternative explanations are plausible, including reciprocal or bidirectional relationships (e.g., procrastination may also exacerbate attentional difficulties and reduce well-being over time). Therefore, findings should be interpreted as statistical associations rather than causal effects. Future longitudinal and experimental studies are needed to clarify temporal precedence and causal mechanisms. Second, although the sample was large and diverse in age, it consisted solely of university students, limiting generalizability to other populations. Third, while our cognitive tasks provided objective measures of executive functioning, they were administered online, which may introduce uncontrolled environmental variability.

Furthermore, the executive tasks assessed broad cognitive constructs but did not capture all relevant components of self-regulation (e.g., planning, error monitoring). Future research could benefit from using neurophysiological measures (e.g., EEG, fNIRS) or ecological momentary assessment techniques to further triangulate findings. Additionally, examining contextual moderators such as task value, deadline proximity, or academic pressure could enrich understanding of when and why procrastination occurs.

### Conclusion

This study offers a comprehensive view of academic procrastination by integrating personality traits, motivation, attention-related difficulties, and behavioral data into a cohesive explanatory model. By bridging subjective tendencies with cognitive performance, it highlights the multifaceted and embedded nature of procrastination as a self-regulatory failure. The indirect effects reported in this study should be understood as statistical, not causal, given the cross-sectional design. These findings nonetheless underscore the importance of combining motivational and executive-level interventions to reduce procrastination and promote academic engagement and psychological well-being.

## Supplementary Materials

The data, analysis scripts, and study materials for this study are available in the Open Science Framework repository (see Picciotto & Fabio, 2025).



PicciottoG.
FabioR. A.
 (2025). From distraction to delay: Unpacking academic procrastination
[Data, scripts, materials]. PsychOpen. https://osf.io/zrab9


## Data Availability

The data, analysis scripts, and study materials for this study are available in the Open Science Framework repository (see Picciotto & Fabio, 2025).
